# Association of common variants in mismatch repair genes and breast cancer susceptibility: a multigene study

**DOI:** 10.1186/1471-2407-9-344

**Published:** 2009-09-25

**Authors:** João Conde, Susana N Silva, Ana P Azevedo, Valdemar Teixeira, Julieta Esperança Pina, José Rueff, Jorge F Gaspar

**Affiliations:** 1 Department of Genetics, Faculty of Medical Sciences, New University of Lisbon. Rua da Junqueira 96. P-1349-008 Lisboa, Portugal; 2 Department of Biochemistry, Faculty of Medical Sciences, New University of Lisbon. Campo dos Mártires da Pátria 130, 1169-056 Lisboa, Portugal; 3 Department of Clinical Pathology, Hospital de S. Francisco Xavier, Estrada do Forte do Alto do Duque, 1495-005 Lisboa, Portugal; 4 Department of Laboratorial Medicine, Faculty of Medical Sciences, New University of Lisbon, Hospital de S. Francisco Xavier, Estrada do Forte do Alto do Duque, 1495-005 Lisboa, Portugal

## Abstract

**Background:**

MMR is responsible for the repair of base-base mismatches and insertion/deletion loops. Besides this, MMR is also associated with an anti-recombination function, suppressing homologous recombination. Losses of heterozygosity and/or microsatellite instability have been detected in a large number of skin samples from breast cancer patients, suggesting a potential role of MMR in breast cancer susceptibility.

**Methods:**

We carried out a hospital-based case-control study in a Caucasian Portuguese population (287 cases and 547 controls) to estimate the susceptibility to non-familial breast cancer associated with some polymorphisms in mismatch repair genes (*MSH3*, *MSH4*, *MSH6*, *MLH1*, *MLH3*, *PMS1 *and *MUTYH*).

**Results:**

Using unconditional logistic regression we found that *MLH3 *(L844P, G>A) polymorphism GA (Leu/Pro) and AA (Pro/Pro) genotypes were associated with a decreased risk: OR = 0.65 (0.45-0.95) (p = 0.03) and OR = 0.62 (0.41-0.94) (p = 0.03), respectively.

Analysis of two-way SNP interaction effects on breast cancer revealed two potential associations to breast cancer susceptibility: *MSH3 *Ala1045Thr/*MSH6 *Gly39Glu - AA/TC [OR = 0.43 (0.21-0.83), p = 0.01] associated with a decreased risk; and *MSH4 *Ala97Thr/*MLH3 *Leu844Pro - AG/AA [OR = 2.35 (1.23-4.49), p = 0.01], GG/AA [OR = 2.11 (1.12-3,98), p = 0.02], and GG/AG [adjusted OR = 1.88 (1.12-3.15), p = 0.02] all associated with an increased risk for breast cancer.

**Conclusion:**

It is possible that some of these common variants in MMR genes contribute significantly to breast cancer susceptibility. However, further studies with a large sample size will be needed to support our results.

## Background

Breast cancer is the first leading cause of cancer mortality in women in the United States and Europe and current estimates suggest that one in eight American women will be diagnosed with breast carcinoma [1]. Various genetic and environmental factors have been established as causes of breast cancer, which is a genetically heterogeneous disease [2-4].

Several studies have identified two major susceptibility genes in breast cancer: *BRCA1 *and *BRCA2 *[5]. These genes have an important role in genome maintenance, in cell-cycle control and in DNA repair in the control of homologous recombination [6,7]. Analysis in families with high risk of breast cancer showed that individuals with point mutations in these genes have a 40-80% of probability to develop breast cancer. However, mutations in these two tumour-suppressor genes account for only 5-10% of all cases of breast cancer [8].

Thus, the challenge is to identify individuals at risk for the remaining sporadic cases. Recent evidence shows that there are probably other background genetic factors that contribute to the development of breast cancer, such as polymorphisms in steroid hormone metabolism and DNA repair pathways that might increase cancer risk [9,10].

Recent evidence that some DNA-repair functions are haploinsufficient adds weight to the idea that variants in DNA-repair genes contribute to cancer risk [10,11]. In fact, higher levels of DNA damage and deficient DNA repair may predispose individuals to cancer [12]. Commonly occurring single nucleotide polymorphisms (SNPs) in DNA repair genes have also been shown to incrementally contribute to cancer risk because of their critical role in maintaining genome integrity [13].

Available evidence indicates that the majority of cancers show instability in specific sequence motifs of dinucleotide repeats. This phenotype of microsatellite instability (MSI) is commonly observed in DNA mismatch repair (MMR) pathway defects [14]. In fact, MSI and/or losses of heterozygosity (LOH) were detected in 83% of skin samples from 12 invasive ductal breast carcinoma patients, suggesting a potential role of MMR in breast cancer susceptibility [15].

Postreplicative mismatch repair (MMR), conserved from prokaryotes to all eukaryotes, including humans, acts on base substitution mismatches and insertion/deletion loops (IDLs) that occur as a result of replication errors that escape the proofreading function of DNA polymerase [16,17]. MMR greatly contributes to the overall fidelity of replication. As such, a decreased activity of MMR confers a mutator phenotype by which the rate of spontaneous mutation is greatly elevated. A characteristic of MMR-deficient cells is instability at microsatellite regions consisting of mono- and di-nucleotide repeats. MSI is a common marker for loss of MMR activity in tumour cells [18].

The main MMR pathway is initiated by the recognition of a mismatch by the heterodimer consisting of the MSH2 and MSH6 proteins (also called MutSα). MutSα is responsible for the recognition of base mismatches and IDLs in mono- to tetranucleotide repeats. This complex, MutSα, is able to recognize most base-base mismatches and short IDLs [19].

Another MMR pathway, consisting of MSH2 and MSH3 heterodimers (MutSβ) is primarily responsible for binding to and correcting insertion/deletion mutations, preferentially dinucleotide and larger IDLs. Upon DNA mismatch recognition the repair process proceeds with the participation of the heterodimer consisting of MLH1 and PMS2 (also called MutLα), which acts as an endonuclease. Subsequent DNA excision is carried out by the exonuclease EXO1 which participates in mismatch-provoked excision directed by strand breaks located either 5' or 3' to the mispair [19,20].

The failure of MMR functions leads to high mutation rates, MSI, LOH, reduction in apoptosis processes and increases in cell survival, as well as predisposition for carcinogenesis [21,22]. MMR is also associated with an anti-recombination function, suppressing homologous recombination and plays a role in DNA-damage signalling [23].

The *MSH2 *gene is central in mismatch recognition and there are some studies reporting mutations [24] and polymorphisms in several *MSH2 *variants [25,26]. However, since there is a scarcity of data about the involvement of polymorphisms in other MMR genes in breast cancer susceptibility, we carried out a hospital-based case-control study in a Caucasian Portuguese population to estimate the potential modifying role of the *MSH3*, *MSH4*, *MSH6*, *MLH1*, *MLH3*, *PMS1 *and *MUTYH *gene polymorphisms on the individual susceptibility to breast cancer.

## Methods

### Study subjects

Healthcare services in Portugal are mainly public and generally assist the whole population, and breast cancer treatment units are located in all the major hospitals. This study includes 287 Caucasian non-familial breast cancer female patients, recruited at São Francisco Xavier Hospital (Department of Laboratorial Medicine) between 2001 and 2005, without previous history of neoplastic disease, thyroid pathology, and blood transfusions. Histological diagnosis was confirmed in all the cases and includes 251 ductal carcinomas (87.4%), 14 lobular carcinomas (4.9%), and 22 cases classified as other type of breast tumours (7.7%). The control population (n = 547) matched for age and ethnicity, with no previous or concurrent malignant disease, was recruited at the same hospital where they were observed for non-malignant pathology. Each case was matched whenever possible with two healthy control individual within the same age and ethnicity groups.

The anonymity of the patients and control population was guaranteed, and all studies were conducted with the informed consent of all the individuals involved. Written informed consent was obtained prior to individual blood withdrawal. Information on demographic characteristics, family history of cancer and lifestyle habits (e.g. smoking, alcohol drinking) was collected using a questionnaire administered by trained interviewers. Former smokers were defined as those who gave up smoking 2 years before cancer diagnosis or 2 years before the inclusion date as corresponding matched case. The response rate was higher than 95% for cases and controls. This study was approved by the ethics board of the involved institutions.

### DNA extraction

Blood samples of all patients and controls were collected into 10 ml ethylenediamine tetracetic acid (EDTA) tubes and stored at -80°C until use. Genomic DNA was obtained from 250 μl of whole blood using a commercially available kit according to the manufacturer's instructions (QIAamp DNA extraction kit; *Qiagen*, Hilden, Germany). Each DNA sample was stored at -20°C until analysis.

### Selection of Polymorphisms

The *MSH3 *(A1045T, A>G, rs26279; R940Q, G>A, rs184967), *MSH4 *(N914S, G>A, rs5745549; A97T, A>G, rs5745325), *MSH6 *(G39E, C>T, rs1042821), *MLH1 *(I219V, A>G, rs1799977), *MLH3 *(L844P, G>A, rs175080), and *MUTYH *(H335Q, G>C, rs3219489) gene polymorphisms are all non-synonymous but one polymorphism, the *PMS1 *(G>C, rs5742933), is a G-to-C transition in the 5' UTR region. All the polymorphisms had a minor allele frequency (MAF) >5%.

### Genotyping

The *MSH3 *(rs26279; rs184967), *MSH4 *(rs5745549; rs5745325), *MSH6 *(rs1042821), *MLH1 *(rs1799977), *MLH3 *(rs175080), *PMS1 *5'UTR (rs5742933) and *MUTYH *(rs3219489) gene polymorphisms were determined by Real-Time PCR using TaqMan^® ^SNP Genotyping Assays from Applied Biosystems (ABI Assays reference: C_800002_1_, C_907914_10, C_1184803_10, C_3286081_10, C_8760558_10, C_1219076_20, C_1082805_10, C_29329633_10 and C_27504565_10, respectively). In order to carry out the allelic discrimination for these polymorphisms the DNA samples were quantified using the Quant-iT™ Picogreen^® ^dsDNA Assay Kit (Invitrogen) according to the manufacturer's recommendations.

The Real-Time PCR amplification was performed in 10 μl reactions containing 10 ng of genomic DNA, 1× SNP Genotyping Assay Mix (containing two primer/probe pairs in each reaction and two fluorescent dye detectors - FAM^® ^and VIC^®^) and 1× TaqMan Universal PCR Master Mix containing the AmpliTaqGold^® ^DNA polymerase, dNTPs and optimized buffer components. The amplification conditions consisted of an initial AmpliTaq Gold^® ^activation at 95°C during 10 min, followed by 40 or more amplification cycles consisting of denaturation at 92°C for 15 sec and annealing/extension at 60°C for 1 min. 10-15% of the genotype determinations were carried out twice in independent experiments with 100% of concordance between experiments.

### Statistical analysis

The Hardy-Weinberg distributions for the different polymorphisms in the control and cancer patients populations in this study were analyzed using exact probability tests available in Mendel (v8.0.1) software [27].

The Chi-Square (χ^2^) test was used to evaluate the differences in genotype frequency, smoking status, and alcohol consumption distributions between cancer patients and controls.

The Kolmogorov-Smirnov test was used in order to verify the normality of the continuous variables (age) and the Levene test was used to analyze the homogeneity of variances. The statistical analysis of the homogeneity of age distributions between cancer patients and controls was carried out using the Student's t-test.

Unconditional multiplicative logistic regression was used to test the global null hypothesis of no association between SNPs and breast cancer, calculating the crude and adjusted odds ratio (ORs), 95% confidence intervals (CIs) and the corresponding p-values. The model for adjusted OR included terms for age at diagnosis (≤ 30, 31-49, 50-69, and ≥ 70 years), the lower age group being the referent class; alcohol consumption (never, social, and regular drinkers), never drinkers being the referent group; and smoking habits (smokers/non-smokers), non-smokers being the referent group. Concerning the analysis of SNP-SNP interactions the genotypic specific risks for two-way SNP interactions were estimated as ORs with associated 95% CIs and p-values. Combined effects between two genotypes were studied by creating different variables, each representing the combination of two genotypes, with the putative "low-risk" homozygous combination as reference category. All the genotype interactions with >5% frequency were considered to be common. Rare interactions were pooled.

All analyses were performed with an SPSS statistical package (version 15) (SPSS Inc., Chicago, IL).

## Results

This study comprised 287 breast cancer patients and 547 healthy controls. The Kolmogorov-Smirnov and the Levene tests showed that the study population follows a normal distribution and homogeneity of the continuous variables (age). The main characteristics (age, smoking habits and alcohol habits) of the case-control populations are listed in Table 1. There were no significant differences between cases and controls concerning age and smoking habits. However, alcohol drinkers are more prevalent in breast cancer patients than in control population (p < 0.001).

**Table 1:** General characteristics for the breast cancer cases (n = 287) and control population (n = 547).

Characteristics	Cases n (%)	Controls n (%)	P value*
**Age a, b**			
≤ 30	1 (0.3%)	2 (0.4%)	0.97 **c**
31-49	66 (23.0%)	119 (21.8%)	
50-69	158 (55.1%)	301 (55.0%)	
≥ 70	62 (21.6%)	125 (22.9%)	
Missing	0	0	
**Smoking habits**			
Never	250 (87.4%)	490 (91.6%)	0.06 **c**
Current	36 (12.6%)	45 (8.4%)	
Missing	1	12	
**Alcohol habits**			
Never	219 (76.3%)	441 (82.6%)	<0.001 **c**
Social	25 (8.7%)	59 (11.0%)	
Regular	43 (15.0%)	34 (6.4%)	
Missing	0	13	
**Histological diagnosis**			
Ductal carcinoma	251 (87.4%)	-	
Lobular carcinoma	14 (4.9%)	-	-
Non-classifiable as ductal or lobular carcinoma	22 (7.7%)	-	

^a ^Age of diagnosis for cases.

^b ^Age of control population at the time of diagnosis for the matched case.

^c ^cases *versus *control group.

* Chi-Square P value [See Methods].

Deviations from Hardy-Weinberg equilibrium (HWE) for the SNPs were examined each in the control population and breast cancer cases. Two SNPs (*MUTYH *- rs3219489 and *MLH1 *- rs1799977) deviated from HWE, only in controls (p = 0.02 and p = 0.04, respectively). These findings are unlikely to be due to genotyping errors because no SNP deviated in both cases and control populations and the allelic discrimination of genotypes for the 9 assays was good.

### Association analysis of SNPs and breast cancer risk

Table 2 presents the genotype frequency, minor allele frequency (MAF) in control population (n = 547) and breast cancer cases (n = 287) and the estimated ORs and 95% CIs, for the 9 successfully genotyped SNPs. There was no difference in genotype frequency between breast cancer cases and controls for all SNPs.

**Table 2:** Genotypic frequencies and minor allele frequency (MAF) in control population (n = 547) and breast cancer cases (n = 287) and analysis of associations of individual SNPs with breast cancer risk.

SNPs	Genotypes	Controls	Cases	MAF		All cases	
		**n (%)**	**n (%)**	**Controls**	**Cases**	**Crude OR (95% CI)**	**Adjusted OR (95% CI) a**

***MSH3 *Ala1045Thr A>G**	**Ala/Ala**	246 (45.2%)	121 (42.3%)			1 (Reference)	1 (Reference)
	**Ala/Thr**	240 (44.1%)	129 (45.1%)	G: 0.33 (± 0.01)	G: 0.35 (± 0.02)	1.09 (0.81-1.48)	1.13 (0.83-1.55)
	**Thr/Thr**	58 (10.7%)	36 (12.6%)			1.26 (0.79-2.02)	1.29 (0.80-2.09)

		**n (%)**	**n (%)**			**Crude OR (95% CI)**	**Adjusted OR (95% CI) a**

***MSH3 *Arg940Gln G>A**	**Arg/Arg**	371 (68.1%)	182 (63.6%)			1 (Reference)	1 (Reference)
	**Arg/Gln**	158 (29.0%)	96 (33.6%)	A: 0.17 (± 0.012)	A: 0.20 (± 0.02)	1.24 (0.91-1.69)	1.23 (0.90-1.68)
	**Gln/Gln**	16 (2.9%)	8 (2.8%)			1.02 (0.43-2.43)	1.03 (0.43-2.47)

		**n (%)**	**n (%)**			**Crude OR (95% CI)**	**Adjusted OR (95% CI) a**

***MSH4 *Asn914Ser G>A**	**Asn/Asn**	496 (91.0%)	263 (91.6%)			1 (Reference)	1 (Reference)
	**Asn/Ser**	49 (9.0%)	23 (8.0%)	A: 0.05 (± 0.01)	A: 0.04 (± 0.01)	0.89 (0.53-1.49)	0.83 (0.49-1.41)
	**Ser/Ser**	0 (0%)	1 (0.3%)			-	-

		**n (%)**	**n (%)**			**Crude OR (95% CI)**	**Adjusted OR (95% CI) a**

***MSH4 *Ala97Thr A>G**	**Ala/Ala**	260 (47.7%)	145 (50.7%)			1 (Reference)	1 (Reference)
	**Ala/Thr**	239 (43.9%)	117 (40.9%)	G: 0.30 (± 0.01)	G: 0.29 (± 0.02)	0.88 (0.65-1.19)	0.85 (0.63-1.16)
	**Thr/Thr**	46 (8.4%)	24 (8.4%)			0.94 (0.55-1.60)	1.03 (0.60-1.79)

		**n (%)**	**n (%)**			**Crude OR (95% CI)**	**Adjusted OR (95% CI) a**

***MSH6 *Gly39Glu C>T**	**Gly/Gly**	354 (65.1%)	195 (68.2%)			1 (Reference)	1 (Reference)
	**Gly/Glu**	174 (32%)	79 (27.6%)	T: 0.19 (± 0.01)	T: 0.18 (± 0.02)	0.82 (0.60-1.13)	0.83 (0.60-1.15)
	**Glu/Glu**	16 (2.9%)	12 (4.2%)			1.36 (0.63-2.94)	1.38 (0.62-3.03)

		**n (%)**	**n (%)**			**Crude OR (95% CI)**	**Adjusted OR (95% CI) a**

***MLH1 *Ile219Val A>G**	**Ile/Ile**	255 (46.7%)	129 (44.9%)			1 (Reference)	1 (Reference)
	**Ile/Val**	251 (46.0%)	129 (44.9%)	G: 0.30 (± 0.01)	G: 0.33 (± 0.02)	1.02 (0.75-1.37)	1.01 (0.74-1.37)
	**Val/Val**	40 (7.3%)	29 (10.1%)			1.43 (0.85-2.42)	1.35 (0.79-2.31)

							

		**n (%)**	**n (%)**			**Crude OR (95% CI)**	**Adjusted OR (95% CI) a**

***MLH3 *Leu844Pro G>A**	**Leu/Leu**	166 (30.5%)	76 (26.6%)			1 (Reference)	1 (Reference)
	**Leu/Pro**	**283 (52%)**	**141 (49.3%)**	A: 0.43 (± 0.02)	A: 0.49 (± 0.02)	**0.69 (0.47-0.99) †**	**0.65 (0.45-0.95) †**
	**Pro/Pro**	**95 (17.5%)**	**69 (24.1%)**			**0.63 (0.42-0.95) †**	**0.62 (0.41-0.94) †**

		**n (%)**	**n (%)**			**Crude OR (95% CI)**	**Adjusted OR (95% CI) a**

***PMS1 *5'UTR Ex1-4 G>C**	**GG**	352 (64.7%)	181 (63.5%)			1 (Reference)	1 (Reference)
	**CG**	179 (32.9%)	90 (31.6%)	C: 0.19 (± 0.01)	C: 0.21 (± 0.02)	0.98 (0.72-1.33)	1.01 (0.74-1.39)
	**CC**	13 (2.4%)	14 (4.9%)			2.09 (0.96-4.55)	1.88 (0.85-4.15)

		**n (%)**	**n (%)**			**Crude OR (95% CI)**	**Adjusted OR (95% CI)a**

***MUTYH *His335Gln**	**His/His**	283 (51.7%)	162 (56.4%)			1 (Reference)	1 (Reference)
**G>C**	**His/Gln**	235 (43.0%)	107 (37.3%)	C: 0.27 (± 0.01)	C: 0.25 (± 0.02)	0.80 (0.59-1.07)	0.810 (0.60-1.10)
	**Gln/Gln**	29 (5.3%)	18 (6.3%)			1.08 (0.58-2.01)	1.053 (0.56-1.98)

^a^ORs were adjusted for: age at diagnosis (≤30, 31-49, 50-69, and ≥70 years), the lower age group being the referent class; alcohol consumption (never, social, and regular drinkers), never drinkers being the referent group; and smoking habits (smokers/non-smokers), non-smokers being the referent group.

^†^Data in bold highlights the statistic significant results (p < 0.05). P values are adjusted by unconditional multiplicative logistic regression.

Regarding the SNP tests for association and the genotype specific risks, obtained by unconditional multiplicative logistic regression, there was no difference in cases and controls for 8 of the 9 SNPs tested (see Table 2). However, one SNP (*MLH3 *- rs175080) showed evidence for association: the homozygous variant (Pro/Pro) was associated with a reduced risk of disease [adjusted OR = 0.62, 95% CI (0.41-0.94), p = 0.03] and the heterozygous (Leu/Pro) was also associated with a protective effect [adjusted OR = 0.65, 95% CI (0.45-0.95), p = 0.03].

### Analysis of two-way SNP interactions

To evaluate the effects in breast cancer susceptibility of combined genotypes between two polymorphisms within the same gene the *MSH3 *and *MSH4 *genotype combination frequencies and the OR values associated with each interaction were estimated [see Additional file 1]. No significant difference was found for these interactions.

The analysis for all SNP-SNP interactions between different genes (with biological plausibility) associated with individual risk for disease was assessed [see Additional file 2]. The data obtained, using unconditional multiplicative logistic regression show that two interactions (*MSH3 *Ala1045Thr/*MSH6 *Gly39Glu and *MSH4 *Ala97Thr/*MLH3 *Leu844Pro; see Table 3) are associated with individual risk for disease. In fact the *MSH3*/*MSH6 *AA/TC are associated with a decreased risk for this pathology (adjusted OR = 0.43, 95% CI (0.21-0.83), p = 0.01). Additionally, three interactions between *MSH4 *Ala97Thr/*MLH3 *Leu844Pro are associated with an increased risk for breast cancer (AG/AA interaction: adjusted OR = 2.35, 95% CI (1.23-4.49), p = 0.01; GG/AA interaction: adjusted OR = 2.11, 95% CI (1.12-3.98), p = 0.02; and GG/AG: adjusted OR = 1.88, 95% CI (1.12-3.15), p = 0.02).

**Table 3:** Two-way SNP interaction effects on breast cancer risk.

Two-way SNP Interactions	Genotypes	Controls	Cases	All cases			
***MSH3 *Ala1045Thr (A>G)/*MSH6 *Gly39Glu (C>T)**		**Controls n (%)**	**Cases n (%)**	**Crude OR (95% CI)**	**P value**	**Adjusted OR (95% CI) a**	**P value**

	**GG/CC**	29 (5.3%)	26 (9.1%)	1 (Reference)	0.36	1 (Reference)	0.26
	**AA/TT** **AG/TT** **GG/TT** **GG/TC**	43 (7.9%)	22 (7.7%)	0.57 (0.27-1.19)	0.14	0.53 (0.25-1.13)	0.10
	**AA/TC**	**81 (14.9%)**	**33 (11.5%)**	**0.45 (0.23-0.89)**	**0.02**	**0.43 (0.21-0.83)**	**0.01**
	**AA/CC**	158 (29.1%)	83 (29.0%)	0.59 (0.32-1.06)	0.08	0.55 (0.30-1.00)	0.05
	**AG/TC**	66 (12.2%)	36 (12.6%)	0.61 (0.31-1.19)	0.14	0.61 (0.310-1.21)	0.16
	**AG/CC**	166 (30.6%)	86 (30.1%)	0.58 (0.32-1.04)	0.07	0.55 (0.30-1.01)	0.05

***MSH4 *Ala97Thr (A>G)/*MLH3 *Leu844Pro (G>A)**		**Controls n (%)**	**Cases n (%)**	**Crude OR (95% CI)**	**P value**	**Adjusted OR (95% CI) a**	**P value**

	**GG/GG**	85 (15.6%)	28 (9.8%)	1 (Reference)	0.02	1 (Reference)	0.01
	**AA/AA** **AA/AG** **AA/GG**	46 (8.5%)	24 (8.4%)	1.58 (0.83-3.04)	0.17	1.70 (0.87-3.32)	0.12
	**AG/AA**	**41 (7.5%)**	**31 (10.8%)**	**2.30 (1.22-4.32)**	**0.01**	**2.35 (1.23-4.49)**	**0.01**
	**AG/AG**	127 (23.3%)	47 (16.4%)	1.12 (0.65-1.93)	0.67	1.03 (0.59-1.78)	0.93
	**AG/GG**	71 (13.1%)	39 (13.6%)	1.67 (0.94-2.98)	0.08	1.54 (0.85-2.78)	0.15
	**GG/AA**	**44 (8.1%)**	**32 (11.2%)**	**2.21 (1.18-4.12)**	**0.01**	**2.11 (1.12-3.98)**	**0.02**
	**GG/AG**	**130 (23.9%)**	**85 (29.7%)**	**1.99 (1.20-3.30)**	**0.01**	**1.88 (1.12-3.15)**	**0.02**

^a ^ORs were adjusted for: age at diagnosis (≤30, 31 -49, 50-69, and ≥70 years), the lower age group being the referent class; alcohol consumption (never, social, and regular drinkers), never drinkers being the referent group; and smoking habits (smokers/non-smokers), non-smokers being the referent group.

Data in bold highlights the statistic significant results. P values are adjusted by unconditional multiplicative logistic regression.

## Discussion

To our knowledge, this is the first comprehensive study to analyze the possible role of 9 common variants of 7 MMR genes and its influence on the genetic susceptibility to breast cancer. Our main focus was to understand the contribution of functionally relevant polymorphisms and SNP-SNP interactions in MMR genes to breast cancer risk. The results hint to a few potential candidate genes and SNP-SNP interactions associated with individual breast cancer susceptibility.

The first plausible association was for *MLH3 *(Leu844Pro) a non-synonymous variant being related with a reduced risk for the homozygous (Pro/Pro) and the heterozygous (Leu/Pro) individuals on breast cancer susceptibility. The *MLH3 *gene has been mainly associated with some cases of HNPCC, where the presence of a considerable number of mutations in this gene is consistent with a possible role in HNPCC progression [28]. Given its role in the repair of IDLs and its involvement in MSI [29], alterations in its structure and function could trigger oncogenic events, such as in breast cancer.

One study on a different cancer population (151 lung cancer cases and 172 hospital controls) by Michielis *et al*. [30] showed that the *MLH3 *(Leu844Pro) polymorphism was associated with an increased risk for lung cancer [OR: 1.97 (1.06-3.65 95% IC), p = 0.04]. However, in contrast to breast cancer, most of lung cancer cases are typically caused by smoking habits, and thus the aetiology of the two cancers is different. In any case, polymorphisms in DNA repair genes may modify cancer risk [31].

Regarding the potential role of the *MLH1 *polymorphism (Ile219Val), it was not associated with breast cancer risk in this study, which is consistent with a previous work conducted by Lee *et al*. [32] that also reports a null association for breast cancer in Korean women (872 cases and 671 controls). Another study published by Smith *et al*. [23] reported a decreased risk of breast cancer [OR = 0.49, 95% CI (0.29-0.85), p < 0.05] in a Caucasian population (336 cases and 416 controls). These differences across studies might be related with different population risk factors and/or with differences in genetic backgrounds.

MMR is achieved by several protein heterodimers. As such, it was important to evaluate a gene-gene interaction model in this study. The analysis of the genotype specific risks shows significant differences for two associations between different MMR genes. The *MSH3 *Ala1045Thr/*MSH6 *Gly39Glu - (AA/TC) interaction was associated with a decreased risk [adjusted OR = 0.43, 95% CI (0.21-0.83), p = 0.01]. It should be noted that the *MSH3 *and *MSH6 *genes act as MMR pathway sensors, detecting errors that occur during DNA replication, being implicated in postreplicative mismatch correction [17].

We also found evidence for an interaction (*MSH4 *Ala97Thr/*MLH3 *Leu844Pro) associated with an increased risk for breast cancer. As described by Santucci-Darmanin *et al*. [33] MLH3 is associated with the meiosis-specific protein MSH4 in mammalian meiotic cells, strongly supporting the possibility that *MLH3 *plays a role in mammalian meiotic recombination [33].

It has been known for some years that the MMR pathway affects the efficiency of meiotic recombination [20]. Nevertheless, MMR proteins are also involved in mitotic recombination processes and play a critical role in maintaining the mitotic stability of eukaryotic genomes [34]. During mitotic recombination MMR proteins prevent exchange between non-identical sequences. In fact, it has been demonstrated that certain homologous sequences recombine only when the MMR pathway is inactive [20]. MMR inactivation leads to an increase in mitotic recombination frequency and an inefficient recombination can increase the risk for cancer susceptibility. Consequently, the anti-recombination function of MMR not only suppresses homologous recombination but also acts to prevent chromosomal rearrangements involving translocations and deletions [22].

As a result, it is possible that structural or function modifications in the *MSH4 *Ala97Thr/*MLH3 *Leu844Pro interaction might be associated with an increased risk for breast cancer, modifying the progression of MMR pathway and therefore increasing the mitotic recombination rates in mammary gland cells. However, larger studies are required to verify the role of *MSH4 *and *MLH3 *genes in mammalian mitotic recombination.

## Conclusion

In summary, we found that the genetic variant of the *MLH3 *gene, Leu844Pro, was associated with decreased risk for breast cancer. Interestingly, an observed decreased risk between common homozygous and heterozygous of the *MSH3 *Ala1045Thr/*MSH6 *Gly39Glu interaction and an increased risk between several combined genotypes of the *MSH4 *Ala97Thr/*MLH3 *Leu844Pro point towards a multiplicative gene-gene interaction model.

The association between cancer risk and several genotypes observed in this study reinforce the hypothesis for the role of the MMR pathway in breast cancer susceptibility. In fact, the MMR plays a key role in maintenance of genomic stability, contributing to tumour suppression by reducing mutations and promoting apoptosis in response to some DNA damage during replication and mitotic recombination processes [17,35]. Different activities and functions of these genes as well as SNP variations may alter the level of repair, leading to higher rates of mutations and therefore an increase of breast cancer risk or conversely play a protective role in breast carcinogenesis. However, independent studies in a larger population are essential to support our results.

## Abbreviations

MMR: mismatch repair; SNP: single nucleotide polymorphism; MSI: microsatellite instability; IDLs: insertion/deletion loops; LOH: loss of heterozygosity; HNPCC: Hereditary Nonpolyposis Colorectal Cancer; HWE: Hardy-Weinberg equilibrium; MAF: minor allele frequency; CI: confidence interval; OR: odds-ratio.

## Competing interests

The authors declare that they have no competing interests.

## Authors' contributions

JC and SNS performed the experiments. APA, VT and JEP participated in the inclusion of patients and controls, and retrieved the information from the patients' records included. JFG and JR were responsible for the study concept and design, and JFG created the database for the study and carried out all of the statistical analyses. JC and SNS wrote the manuscript and JFG and JR also contributed to writing the manuscript. The manuscript had been read by all authors.

## Pre-publication history

The pre-publication history for this paper can be accessed here:

http://www.biomedcentral.com/1471-2407/9/344/prepub

## Supplementary Material

Additional file 1 **Effects in breast cancer susceptibility of combined genotypes between two polymorphisms within the same gene**. The table provided depicts the genotype combination frequencies and the OR values between two polymorphisms within the same gene - MSH3 and MSH4. Click here for file

Additional file 2 **All two-way SNP interaction effects on breast cancer risk**. The data supplied represents the frequencies and the OR values for all SNP-SNP interactions between different genes associated with individual risk for breast cancer. Click here for file
