# Supplementary material for: Association of common variants in mismatch repair genes and breast cancer susceptibility: a multigene study
Source: BMC Cancer. 2009 Sep 25;9:344. doi: 10.1186/1471-2407-9-344 (PMC2761943; doi:10.1186/1471-2407-9-344)
Supplement: Additional file 1 — Effects in breast cancer susceptibility of combined genotypes between two polymorphisms within the same gene. The table provided depicts the genotype combination frequencies and the OR values between two polymorphisms within the same gene - MSH3 and MSH4. [file 1471-2407-9-344-S1.PDF]

Additional file 1 – Effects in breast cancer susceptibility of combined genotypes between two polymorphisms within the same gene.

Interactions	Genotypes	Controls	Cases	All cases			
MSH3 Ala1045Thr (A>G) / *MSH3* Arg940Gln (G>A)		n (%)	n (%)	Crude OR (95% CI)	P value	Adjusted OR (95% CI) [a]	P value
	AA/GG	246 (45.2%)	120 (42.0%)	1 (Reference)	0.56	1 (Reference)	0.63
	AA/AG<br>AG/AA<br>GG/GG<br>GG/AA	24 (4.4%)	17 (5.9%)	1.45 (0.75-2.81)	0.27	1.47 (0.75-2.87)	0.26
	AG/GG	116 (21.3%)	54 (18.9%)	0.95 (0.65-1.41)	0.81	1.03 (0.69-1.53)	0.88
	AG/AG	123 (22.6%)	75 (26.2%)	1.25 (0.87-1.79)	0.23	1.26 (0.87-1.82)	0.23
	GG/AG	35 (6.4%)	20 (7.0%)	1.17 (0.65-2.12)	0.60	1.22 (0.67-2.24)	0.52
MSH4 Asn914Ser (G>A) / *MSH4* Ala97Thr (A>G)		Controls n (%)	Cases n (%)	Crude OR (95% CI)	P value	Adjusted OR (95% CI) [a]	P value
	GG/GG	230 (42.2%)	133 (46.5%)	1 (Reference)	0.56	1 (Reference)	0.46
	AA/GG<br>AG/AG	19 (3.5%)	13 (4.5%)	1.18 (0.57-2.47)	0.66	1.12 (0.53-2.38)	0.77
	AG/GG	30 (5.5%)	11 (3.8%)	0.63 (0.31-1.31)	0.22	0.60 (0.29-1.25)	0.17
	GG/AA	46 (8.4%)	24 (8.4%)	0.90 (0.53-1.55)	0.71	0.99 (0.57-1.72)	0.98
	GG/AG	220 (40.4%)	105 (36.7%)	0.83 (0.60-1.13)	0.23	0.80 (0.58-1.11)	0.18

[a] ORs were adjusted for: age at diagnosis (≤30, 31 –49, 50–69, and ≥70 years), the lower age group being the referent class; alcohol consumption (never, social, and regular drinkers), never drinkers being the referent group; and smoking habits (smokers/non-smokers), non-smokers being the referent group.

P values are adjusted by unconditional multiplicative logistic regression.
