# Supplementary material for: Association of common variants in mismatch repair genes and breast cancer susceptibility: a multigene study
Source: BMC Cancer. 2009 Sep 25;9:344. doi: 10.1186/1471-2407-9-344 (PMC2761943; doi:10.1186/1471-2407-9-344)
Supplement: Additional file 2 — All two-way SNP interaction effects on breast cancer risk. The data supplied represents the frequencies and the OR values for all SNP-SNP interactions between different genes associated with individual risk for breast cancer. [file 1471-2407-9-344-S2.PDF]

Additional file 2 – All two-way SNP interaction effects on breast cancer risk.

Two-way SNP Interactions	Genotypes	Controls	Cases	All cases			
***MLH1* Ile219Val (A>G) / *PMS1* 5'UTR Ex1-4(G>C)**		**Controls n (%)**	**Cases n (%)**	**Crude OR (95% CI)**	**P value**	**Adjusted OR (95% CI) [a]**	**P value**
	**GG/GG**	24 (4.4%)	18 (6.3%)	1 (Reference)	0.27	1 (Reference)	0.26
	**AACC<br>AG/CC<br>GG/CC<br>GG/CG**	28 (5.1%)	23 (8.1%)	1.10 (0.48-2.50)	0.83	1.23 (0.53-2.86)	0.63
	**AA/CG**	87 (16.0%)	48 (16.8%)	0.74 (0.36-1.49)	0.39	0.89 (0.43-1.83)	0.74
	**AA/GG**	162 (29.8%)	72 (25.3%)	0.59 (0.30-1.16)	0.13	0.66 (0.33-1.31)	0.23
	**AG/CG**	77 (14.2%)	33 (11.6%)	0.57 (0.27-1.19)	0.14	0.61 (0.29-1.30)	0.20
	**AG/GG**	166 (30.5%)	91 (31.9%)	0.73 (0.38-1.42)	0.35	0.84 (0.43-1.66)	0.62
***MLH1* Ile219Val (A>G) / *MLH3* Leu844Pro (G>A)**		**Controls n (%)**	**Cases n (%)**	**Crude OR (95% CI)**	**P value**	**Adjusted OR (95% CI) [a]**	**P value**
	**AA/AA**	48 (8.8%)	29 (10.1%)	1 (Reference)	0.18	1 (Reference)	0.19
	**AA/AG**	128 (23.5%)	69 (24.1%)	0.89 (0.52-1.54)	0.68	0.83 (0.47-1.44)	0.50
	**AA/GG**	78 (14.3%)	30 (10.5%)	0.68 (0.34-1.19)	0.16	0.60 (0.32-1.14)	0.12
	**AG/AA**	40 (7.4%)	32 (11.2%)	1.32 (0.69-2.55)	0.40	1.27 (0.65-2.48)	0.48
	**AG/AG**	131 (24.1%)	57 (19.9%)	0.72 (0.41-1.26)	0.25	0.66 (0.38-1.17)	0.15
	**AG/GG**	79 (14.5%)	40 (14.0%)	0.84 (0.46-1.52)	0.56	0.80 (0.43-1.47)	0.47
	**GG/AA<br>GG/AG<br>GG/GG**	40 (7.4%)	29 (10.1%)	1.20 (0.62-2.33)	0.59	1.07 (0.54-2.101)	0.85

Additional file 2 (Cont.) - All two-way SNP interaction effects on breast cancer risk.

Two-way SNP Interactions	Genotypes	Controls	Cases	All cases			
***MSH3* Ala1045Thr (A>G) / *MSH6* Gly39Glu (C>T)**		**Controls n (%)**	**Cases n (%)**	**Crude OR (95% CI)**	**P value**	**Adjusted OR (95% CI) [a]**	**P value**
	**GG/CC**	29 (5.3%)	26 (9.1%)	1 (Reference)	0.36	1 (Reference)	0.26
	**AA/TT<br>AG/TT<br>GG/TT<br>GG/TC**	43 (7.9%)	22 (7.7%)	0.57 (0.27-1.19)	0.14	0.53 (0.25-1.13)	0.10
	**AA/TC**	81 (14.9%)	33 (11.5%)	**0.45 (0.23-0.89)**	**0.02**	**0.43 (0.21-0.83)**	**0.01**
	**AA/CC**	158 (29.1%)	83 (29.0%)	0.59 (0.32-1.06)	0.07	0.55 (0.30-1.00)	0.05
	**AG/TC**	66 (12.2%)	36 (12.6%)	0.61 (0.31-1.19)	0.14	0.61 (0.31-1.21)	0.16
	**AG/CC**	166 (30.6%)	86 (30.1%)	0.58 (0.32-1.04)	0.07	0.55 (0.30-1.01)	0.05
***MSH3* Arg940Gln (G>A) / *MSH6* Gly39Glu (C>T)**		**Controls n (%)**	**Cases n (%)**	**Crude OR (95% CI)**	**P value**	**Adjusted OR (95% CI) [a]**	**P value**
	**AG/CC**	98 (18.0%)	60 (21.0%)	1 (Reference)	0.59	1 (Reference)	0.62
	**GG/TT<br>AG/TT<br>AA/TC<br>AA/CC**	32 (5.9%)	20 (7.0%)	1.02 (0.54-1.95)	0.95	1.02 (0.54-2.00)	0.92
	**GG/TC**	113 (20.8%)	48 (16.8%)	0.69 (0.44-1.11)	0.12	0.70 (0.44-1.13)	0.14
	**GG/CC**	246 (45.2%)	128 (44.8%)	0.85 (0.58-1.25)	0.41	0.86 (0.58-1.27)	0.45
	**AG/TC**	55 (10.1%)	30 (10.5%)	0.89 (0.52-1.54)	0.68	0.92 (0.52-1.60)	0.75

Additional file 2 (Cont.) - All two-way SNP interaction effects on breast cancer risk.

Two-way SNP Interactions	Genotypes	Controls	Cases	All cases			
***MUTYH* His335Gln (G>C) / *MSH6* Gly39Glu (C>T)**		**Controls n (%)**	**Cases n (%)**	**Crude OR (95% CI)**	**P value**	**Adjusted OR (95% CI) [a]**	**P value**
	**CG/CC**	163 (30.0%)	69 (24.1%)	1 (Reference)	0.15	1 (Reference)	0.26
	**GG/TT<br>CG/TT<br>CC/TC<br>CC/CC**	45 (8.3%)	30 (10.5%)	1.58 (0.92-2.71)	0.10	1.50 (0.86-2.60)	0.15
	**GG/TC**	92 (16.9%)	44 (15.4%)	1.13 (0.72-1.78)	0.60	1.11 (0.69-1.76)	0.67
	**GG/CC**	175 (32.2%)	112 (39.2%)	1.51 (1.05-2.19)	0.03	1.44 (0.99-2.10)	0.06
	**CG/TC**	69 (12.7%)	31 (10.8%)	1.06 (0.64-1.77)	0.82	1.02 (0.61-1.71)	0.94
***MSH4* Asn914Ser (G>A) / *MLH3* Leu844Pro (G>A)**		**Controls n (%)**	**Cases n (%)**	**Crude OR (95% CI)**	**P value**	**Adjusted OR (95% CI) [a]**	**P value**
	**GG/GG**	152 (27.9%)	73 (25.5%)	1 (Reference)	0.28	1 (Reference)	0.17
	**AA/AG<br>AG/AA<br>AG/GG**	22 (4.0%)	16 (5.6%)	1.51 (0.75-3.06)	0.25	1.50 (0.74-3.06)	0.26
	**AG/AG**	27 (5.0%)	8 (2.8%)	0.62 (0.27-1.43)	0.26	0.54 (0.23-1.28)	0.16
	**GG/AA**	87 (16.0%)	57 (19.9%)	1.36 (0.88-2.11)	0.16	1.41 (0.90-2.20)	0.13
	**GG/AG**	256 (47.1%)	132 (46.2%)	1.07 (0.76-1.52)	0.69	1.05 (0.73-1.49)	0.81

Additional file 2 (Cont.) – All two-way SNP interaction effects on breast cancer risk.

Two-way SNP Interactions	Genotypes	Controls	Cases	All cases			
***MSH4* Ala97Thr (A>G) / *MLH3* Leu844Pro (G>A)**		**Controls n (%)**	**Cases n (%)**	**Crude OR (95% CI)**	**P value**	**Adjusted OR (95% CI) [a]**	**P value**
	**GG/GG**	85 (15.6%)	28 (9.8%)	1 (Reference)	0.02	1 (Reference)	0.01
	**AA/AA<br>AA/AG<br>AA/GG**	46 (8.5%)	24 (8.4%)	1.58 (0.83-3.04)	0.17	1.70 (0.87-3.32)	0.12
	**AG/AA**	41 (7.5%)	31 (10.8%)	**2.30(1.22-4.32)**	**0.01**	**2.35 (1.23-4.49)**	**0.01**
	**AG/AG**	127 (23.3%)	47 (16.4%)	1.12 (0.65-1.93)	0.67	1.03 (0.59-1.78)	0.93
	**AG/GG**	71 (13.1%)	39 (13.6%)	1.67 (0.94-2.98)	0.08	1.54 (0.85-2.78)	0.15
	**GG/AA**	44 (8.1%)	32 (11.2%)	**2.21 (1.18-4.12)**	**0.01**	**2.11 (1.12-3.98)**	**0.02**
	**GG/AG**	130 (23.9%)	85 (29.7%)	**1.99 (1.20-3.30)**	**0.01**	**1.88 (1.12-3.15)**	**0.02**
***MLH1* Ile219Val (A>G) / *MSH4* Asn914Ser (G>A)**		**Controls n (%)**	**Cases n (%)**	**Crude OR (95% CI)**	**P value**	**Adjusted OR (95% CI) [a]**	**P value**
	**GG/GG**	34 (6.2%)	27 (9.4%)	1 (Reference)	0.36	1 (Reference)	0.49
	**AA/AA<br>AA/AG<br>AG/AG<br>GG/AG**	49 (9.0%)	24 (8.4%)	0.62 (0.31-1.25)	0.18	0.63 (0.30-1.28)	0.20
	**AA/GG**	236 (43.3%)	114 (39.7%)	0.61 (0.35-1.06)	0.08	0.66 (0.37-1.16)	0.15
	**AG/GG**	226 (41.5%)	122 (42.5%)	0.68 (0.39-1.18)	0.17	0.73 (0.41-1.29)	0.27

Additional file 2 (Cont.) – All two-way SNP interaction effects on breast cancer risk.

Two-way SNP Interactions	Genotypes	Controls	Cases	All cases			
MLH1 Ile219Val (A>G) / *MSH4* Ala97Thr (A>G)		Controls n (%)	Cases n (%)	Crude OR (95% CI)	P value	Adjusted OR (95% CI) [a]	P value
	**AG/GG**	121 (22.2%)	59 (20.6%)	1 (Reference)	0.29	1 (Reference)	0.19
	**AA/AA AG/AA GG/AA GG/AG GG/GG**	81 (14.9%)	48 (16.8%)	1.22 (0.76-1.95)	0.42	1.29 (0.79-2.10)	0.31
	**AA/AG**	116 (21.3%)	45 (15.7%)	0.80 (0.50-1.27)	0.33	0.79 (0.49-1.27)	0.32
	**AA/GG**	118 (21.7%)	73 (25.5%)	1.27 (0.83-1.94)	0.27	1.32 (0.85-2.04)	0.21
	**AG/AG**	109 (20.0%)	61 (21.3%)	1.15 (0.74-1.79)	0.54	1.17(0.74-1.83)	0.50

[a] ORs were adjusted for: age at diagnosis (≤30, 31 –49, 50–69, and ≥70 years), the lower age group being the referent class; alcohol consumption (never, social, and regular drinkers), never drinkers being the referent group; and smoking habits (smokers/non-smokers), non-smokers being the referent group.

Data in bold highlights the statistic significant results. P values are adjusted by unconditional multiplicative logistic regression.
